# Development of a psychological intervention for fatigue after stroke

**DOI:** 10.1371/journal.pone.0183286

**Published:** 2017-08-17

**Authors:** Simiao Wu, Trudie Chalder, Kirstin E. Anderson, David Gillespie, Malcolm R. Macleod, Gillian E. Mead

**Affiliations:** 1 Department of Neurology, West China Hospital, Sichuan University, Chengdu, China; 2 Centre for Clinical Brain Sciences, University of Edinburgh, Edinburgh, United Kingdom; 3 Department of Psychological Medicine, Kings College London, London, United Kingdom; 4 Department of Geriatric Medicine, University of Edinburgh, Edinburgh, United Kingdom; 5 Division of Clinical Neurosciences, University of Edinburgh, Edinburgh, United Kingdom; Kessler Foundation, UNITED STATES

## Abstract

**Background and aim:**

Post-stroke fatigue (PSF) is common and distressing, but there is insufficient evidence to recommend any effective treatment for it. Psychological interventions are effective in treating fatigue in other conditions. This paper describes the development and evaluation of the feasibility of a psychological intervention for PSF.

**Methods:**

Based on psychological correlates of PSF and evidence-based psychological interventions for fatigue in other medical conditions, we developed a manualised psychological intervention for PSF, with input from stroke clinicians, psychological therapists, and stroke survivors. The intervention was delivered by a clinical psychologist to 12 participants with PSF to test its acceptability and feasibility. According to the feedback from participants and therapists, the intervention was refined for future use.

**Results:**

The intervention consisted of six individual, face-to-face treatment sessions, and one follow-up, telephone-delivered booster session. It included psycho-education and discussion of strategies to promote physical and social activities and to challenge unhelpful thoughts. Four participants dropped out and the remaining eight participants completed the intervention. These eight participants also completed all assessments and feedback and reported fatigue levels as lower at the end of the study than at the baseline. All participants reported favourable opinions on the intervention and suggested that the last two treatment sessions be combined and the booster session be delivered in person as opposed to telephone.

**Conclusions:**

This psychological intervention was acceptable to stroke patients and was feasible in the local health service. These findings suggest that a randomised controlled trial to test efficacy is warranted.

## Introduction

Post-stroke fatigue (PSF) is a common and distressing problem. It impedes patients’ participation in daily activities and stroke rehabilitation [1] and is associated with a higher risk of institutionalisation and death [2]. The mechanisms of PSF are elusive. Psychological factors are the most commonly reported associations of PSF. Although randomised controlled trials (RCTs) have demonstrated the efficacy of psychological interventions in treating fatigue in other conditions such as cancer-related fatigue [3] and chronic fatigue syndrome [4], there is insufficient evidence to recommend any effective treatment for PSF as reported in a recent Cochrane review of interventions for post-stroke fatigue [5]. Given that the psychological profile of patients with PSF is comparable to patients with these other conditions [6], psychological interventions are promising to treat PSF.

A Dutch study which tested a psychological intervention for PSF reported that a combination of the psychological intervention with graded activity training was superior to the psychological intervention alone in reducing PSF [7]. In addition, patients assigned to a waiting-list control condition showed no significant change in fatigue scores whilst time effect on reduction in fatigue scores was evident in both psychological intervention group and psychological plus physical intervention group. However, as there is no control simultaneously observed with two intervention groups, we could not exclude the ‘placebo’ effect. Furthermore, this psychological intervention was delivered by neuropsychologists, whilst due to the constrained resources within the UK National Health System (NHS), it is not practical to provide this psychologist-delivery approach to every patient with PSF within NHS or countries with similar health systems.

Therefore, the aim of this study was to develop a straightforward and cost-effective psychological intervention for PSF, which would be suitable for delivery by more general medical staff who provide stroke care, such as stroke nurses, so that it could be affordable to the NHS. Psychological interventions are ‘complex interventions’ that consist of multiple therapeutic components. The UK Medical Research Council (MRC) has suggested a phased and iterative framework for the development and evaluation of complex interventions [8]. The current study was related to the development phase (phase 1) and part of the feasibility phase (phase 2) of this framework [8].

## Methods

**Fig 1:**
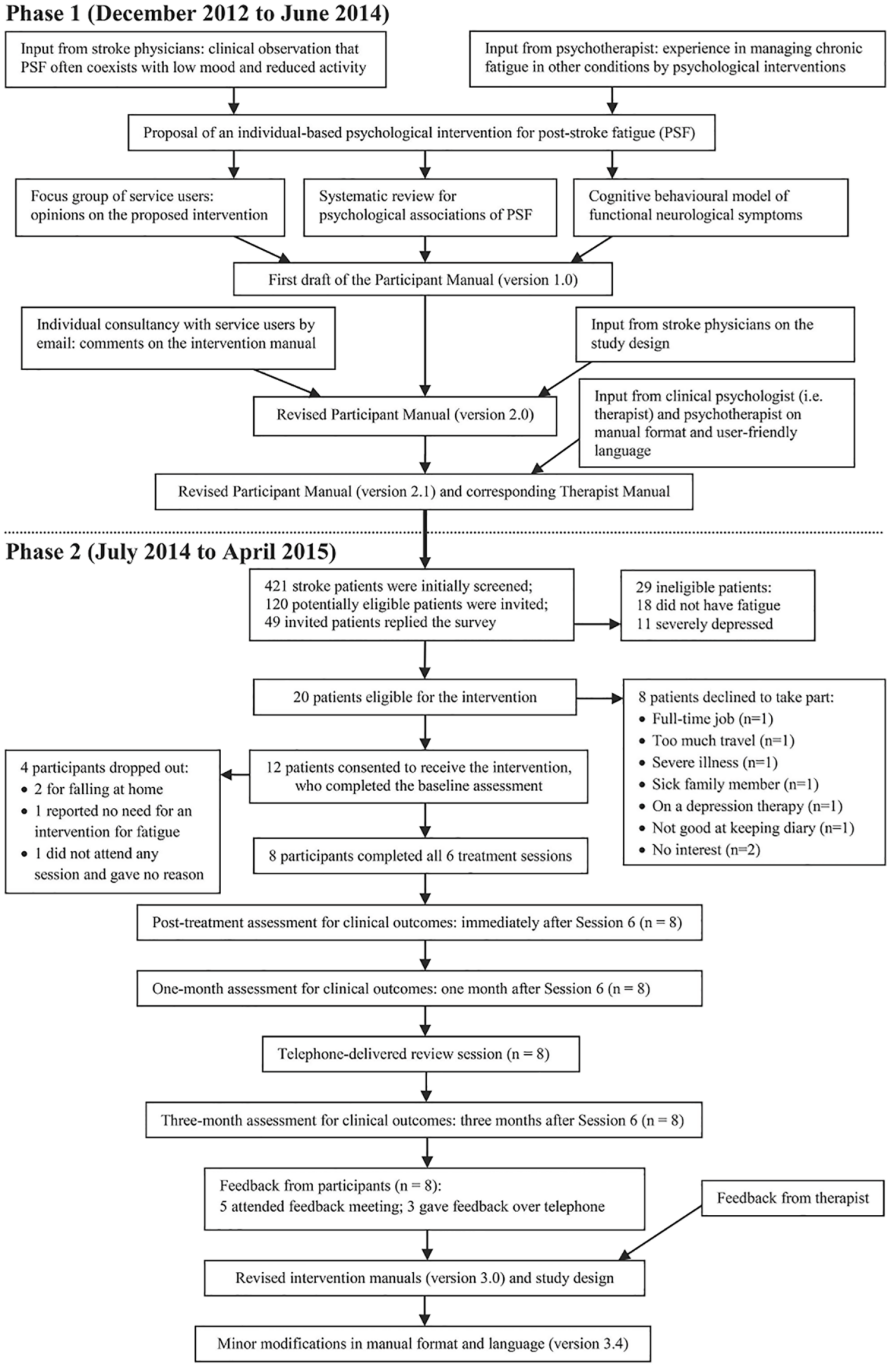
Flowchart of the development and feasibility study of a psychological intervention for post-stroke fatigue.

The intervention was developed by a multidisciplinary group of stroke clinicians (SW, GM, MM), clinical psychologists (KA, DG), a cognitive behavioural psychotherapist (TC), and stroke survivors and carers. Fig 1 presents an overview of the study process.

### Phase 1: Developing the intervention programme

#### Proposal of a psychological intervention

Based on the stroke clinicians’ (GM, MM) clinical observation that PSF often coexists with low mood and reduced physical activity and the psychotherapist’s (TC) experience in the management of chronic fatigue, we proposed a psychological intervention to treat PSF by targeting patients’ thoughts and behaviours. We discussed the concept of this intervention with a focus group of service users (10 stroke survivors and five carers, from the Yorkshire Stroke Research Network) for their preliminary opinions on its acceptability. The users shared their experience of PSF and considered that the proposed intervention would be acceptable.

#### Identifying theoretical evidence

In order to identify the intervention targets in a broader context of stroke illness, we conducted a systematic review to explore the psychological correlates of PSF [9]. Drawing on the evidence from this literature and with input from stroke physicians and clinical psychologists, we developed a stroke-specific model of PSF, which suggested that depressive symptoms, anxiety, lower self-efficacy, passive coping, reduced physical activity, sleeping problems, and inadequate social support were associated with PSF [10].

#### Developing treatment rationale

These interacting psychological, behavioural and environmental factors can be understood within a cognitive behavioural model for neurological functional symptoms [11], which act to perpetuate fatigue symptoms and disability. The premise of cognitive behavioural therapy (CBT) is that changing unhelpful thoughts and behaviours impacts on how people feel physically and emotionally [12]. We hypothesised that a gradual increase in physical activity would challenge any unhelpful beliefs that stop patients with PSF from doing things, by which the vicious cycle of PSF would be interrupted. Patients’ self-efficacy would then be strengthened and a reduction in fatigue and an improvement in physical activity would be achieved. In order to engage the patient in this process, the potential benefits of the approach were explained. Patients were told that PSF was reversible and they were encouraged to overcome the fear of taking physical activity (i.e. the cognitive approach of this intervention), and were encouraged to promote a balance between daily activities, rest and sleep and then gradually increase their level of physical activity (i.e. the behavioural approach of this intervention).

#### Drafting intervention manuals

We adapted the intervention from a nurse-delivered behaviourally-oriented intervention for cancer-related fatigue [3] and a self-management cognitive behavioural approach for chronic fatigue syndrome [13]. We drafted a Participant Manual to provide an outline of each session. The provisional programme consisted of three two-hour sessions with two-week intervals between sessions.

We distributed the manual by email to a user group (five stroke survivors, from the Scottish Stroke Research Network) for their opinions on the manual’s content and format. Following the user feedback, we extended the intervention programme to six treatment sessions plus one booster session at one month after session 6, and halved the length of each session to one hour. We modified the Participant Manual to a more user-friendly version and developed a corresponding Therapist Manual.

#### Determining measures for clinical outcomes

Consistent with the recommendation for assessing functional recovery after stroke [14], we considered three criteria in selecting outcome measures: a) fitting the framework of the International Classification of Functioning, Disability, and Health [15]; b) good psychometric properties in stroke patients; and c) feasible for postal delivery. We measured the presence of fatigue by a case definition of PSF [16], fatigue severity by Fatigue Assessment Scale [17], depression severity by Patient Health Questionnaire-9 (PHQ-9) [18], independence by Nottingham Extended Activities of Daily Living (NEADL) [19], and stroke-specific quality of life by Stroke Impact Scale (SIS) 3.0 [20].

### Phase 2: A feasibility study

We conducted a single-arm feasibility study for this intervention. Ethical approval was granted by the South East Scotland Research Ethics Committee (14/SS/0093). Separate written consent was obtained from participants for the screening stage and for the intervention stage. The study was registered at https://clinicaltrials.gov/ (NCT02131532). The authors confirmed that all ongoing and related trials for this intervention were registered.

#### Participants

Inclusion criteria: a) ≥18 years old, b) stroke 3–24 months previously, c) self-reported fatigue, and d) living in the Lothian area, Scotland, UK. Exclusion criteria: a) severe depression (assessed by the screening questionnaire PHQ-9 scored 15 or more), b) significant impairments in cognition or communication (as recorded in medical notes or assessed by responsible stroke physicians or general practitioners who had helped us identify potential participants), c) medically unstable or living in nursing home, or d) currently in other research studies or receiving any treatment for fatigue or depression, or would increase the physical burden of the participants.

Sample size calculation is not normally required for feasibility or pilot studies [21]. Some researchers suggested that a sample of 10 or fewer would be sufficient for studies assessing acceptability of formatting or ease of administration of an instrument [22]. Such a small group of participants had been used in the development and preliminary evaluation of a cognitive behavioural approach to manage fatigue in patients with multiple sclerosis [23], which successfully informed the design of a multi-centre RCT [24]. Thus we pre-specified a sample size of 12 for this feasibility study to allow for two dropouts. This sample size was anticipated to be achievable in the limited time (three to four months) available for the recruitment in this feasibility study.

#### Screening stage

From July 2014 to October 2014, we identified potential participants from patients who had been discharged from a stroke unit (Royal Infirmary of Edinburgh), patients who had visited an outpatient stroke clinic (Western General Hospital), and those who had been visited by nurses from a community stroke service (Chest Heart & Stroke Scotland, CHSS) in Lothian area, Scotland. We reviewed patients’ medical records and contacted their General Practitioners or responsible physicians for their eligibility. Only patients with adequate cognitive and language capabilities would be referred by their doctors for current study. We approached potentially eligible patients by posting two questionnaires: a single question for fatigue and the PHQ-9 for depression, together with an information sheet and a consent form, to each potential participant. Patients who answered ‘yes’ to the fatigue question and had a PHQ-9 score of 14 or less (i.e. no severe depression), were mailed another information sheet for the intervention and invited to meet with the principal investigator (SW) to sign the consent form at the Clinical Research Facility of Royal Infirmary of Edinburgh. For patients with a PHQ-9 score between 10 and 14 (indicating moderate depression), we wrote to them to inform them that they potentially had some symptoms of depression, and suggested that they either take part in the current study or consult their General Practitioners for further advice on depression. Only patients who chose to receive the current intervention and did not receive any other interventions for fatigue or depression were included.

At the meeting in preparation for requesting participants’ consent for inclusion in the study, the researcher (SW) together with a stroke research nurse further assessed the cognitive abilities of potential participants, and patients were included if they were assessed by both the researcher and the nurse as of adequate cognitive capabilities in verbal communication and completing reading and writing tasks.

#### Intervention stage

In the long term, we envisage this intervention to be delivered by stroke nurses. However, we decided a clinical psychologist (KA) should test the feasibility in the current study. If we had tested feasibility with nurses and it hadn’t worked we would not have known whether it was the intervention that hadn’t worked or the person delivering it. KA was supervised by another clinical psychologist (DG). Both psychologists were experienced in the psychological treatment of stroke patients. The intervention was delivered individually to each participant at the Department of Clinical Psychology of a rehabilitation hospital (Astley Ainslie Hospital, Edinburgh). A checklist of key therapeutic components was provided for the therapist to read prior to the delivery of each session and to complete after the session.

#### Outcome measures

Feasibility measures were primary outcomes for this study. We recorded the number of eligible patients at each stage of recruitment and reasons for ineligibility, the number of dropouts, reasons for withdrawal and whether the sessions were attended as planned. After all retained participants completed their sessions and assessments, they were invited to a focus group and were asked to complete an anonymised feedback questionnaire, regarding the usefulness of the intervention, difficulty in completing homework tasks, adequacy of session arrangement, and modes of delivery. Participants who were unable to attend the focus group were invited to give feedback by telephone. The therapist provided written feedback on the acceptability and feasibility of the intervention.

Clinical measures were secondary outcomes for this study. Baseline assessments were completed by all participants at the consenting meeting. Three post-treatment assessments (immediately after Session 6, one month later, and three months later) were completed by participants who completed all sessions (i.e. the retained participants) by postal questionnaires. The three-month follow-up for the final participant was completed in April 2015.

#### Statistical analysis

Statistical analyses were performed in IBM SPSS (version 21). We performed descriptive analyses for feasibility outcomes. For clinical outcomes, we tested the assumption of normality of distribution by Shapiro-Wilk test. We used the independent t-test and the Fisher’s exact test for the comparisons between the retained participants and those who dropped out. For the retained participants, we compared their outcomes (mean values or proportions) between baseline assessment and each of the post-treatment assessments, using the paired t-tests and the McNemar test. Considering the correction for multiple comparisons, the critical level of statistical significance would drop from 0.05 to 0.001 (2-tailed). However, there might be interactions between some measures and the results of post-treatment assessments were not independent of each other. Thus the appropriate adjusted critical significance level might be somewhere between 0.001 and 0.05, but the exact value was unknown. Therefore, for each outcome, we reported the mean difference of scores between baseline and each post-treatment assessment, with relevant 95% confidence intervals and *p* values.

## Results

### Phase 1

#### Intervention programme

Table 1 summarises the structure and content of the intervention. The duration of each session was one hour. Session 1 had an extra 30 minutes to allow for the development of a collaborative therapeutic relationship. There were two-week intervals between sessions. Homework tasks (e.g. keeping a diary and increasing daily activities) were negotiated during sessions for participants to complete at home. One month after the final treatment session, the therapist delivered a booster session to each participant by telephone.

**Table 1:** Structure and content of intervention programme.

Sessions	Cognitive strategies	Behavioural strategies
Session 1: Introduction and psychoeducation	Education about PSF: Reassure the participant that impact of PSF is reversible	Activity and sleep diaries: Identify targets to facilitate the improvement of PSF
Session 2: Goal setting and activity planning	Goal setting: Set goals to develop a balance between daily activities, rest and sleep	Activity planning: Divide goals into small and manageable steps; specify activities to work toward the goals
Session 3: Progress assessment and goal modification	Goal modification: Modify goals according to participant’s progress and, where applicable, gradually increase participants’ activity levels	Activity rescheduling: Adjust activity plan according to the new goals
Session 4: Cognitive restructuring	Challenging unhelpful thoughts: Identify participant’s thoughts about PSF that invoke unpleasant emotions and relevant behavioural responses; discuss alternative, more positive and realistic thoughts	Acting against unhelpful thoughts: Foster behavioural changes according to the alternative thoughts
Session 5: Dealing with blocks and setbacks	Identifying factors that block progress: Discuss potential blocks to progress; reinforce the challenge of unhelpful thoughts	Managing blocks and setbacks: Take action to overcome factors that block progress
Session 6: Overview and future planning	Review of learned skills; summary of achieved goals; making future plans	Providing worksheets for future use
Booster session	Reviewing the progress in overcoming fatigue in the past month; negotiating plans to make further improvement	

### Phase 2

#### Recruitment

From July 2014 to October 2014, we screened 421 patients (who had had a stroke in the past three months to two years) and sent invitation letters to 120 potentially eligible patients. Forty-nine eligible patients completed questionnaires, of whom 31 reported fatigue by the single question and the other 18 did not. Of the 31 fatigued patients, 11 had a PHQ-9 score of 15 or more (indicating severe depression) and so were excluded; the remaining 20 patients were eligible for the intervention, of whom eight declined to receive it (reasons for decline see Fig 1).

Twelve eligible patients (five women and seven men, all ischaemic stroke) consented to receive the intervention, with a mean age of 63 years (range 47 to 85). Eight participants had had their first-ever stroke and the other four recurrent stroke in the past two years. The mean duration from the recent stroke to recruitment was 16 months (range 5 to 23 months). All 12 participants were allocated to receive the intervention.

#### Retention

Four participants (all women) dropped out: one participant withdrew after the first session as, following discussion with the therapist, she explained that she did not desire an intervention for her fatigue but that her chief purpose in participation had been to contribute to the research; a second participant withdrew after the initial session and the third participant after the second session because of unrelated physical ill-health problems; the fourth participant failed to attend any of the sessions despite reminders and gave no reasons.

The remaining eight participants (one woman and seven men) completed all sessions and all post-treatment assessments. The three-month follow-up assessment for the final participant was completed in April 2015. Women were more likely to drop out (*p* = 0.01) as were those with lower scores of physical strength in SIS (*p* = 0.03). There were no differences between groups in age, time since stroke or other baseline outcomes (Table 2).

**Table 2:** Clinical characteristics of participants at recruitment.

Characteristics	Participants completing all sessions (n = 8)	Participants dropping out (n = 4)	*p* values
Female/Male	1/7	4/0	0.01*
Mean age (years)	62.0 (SD = 14.7)	64.5 (SD = 8.8)	0.76
First/Recurrent stroke	6/2	2/2	0.55
Mean time since recent stroke (months)	16.3 (SD = 5.1)	14.8 (SD = 7.5)	0.69
Meet the case definition of post-stroke fatigue	5 (62.5%)	3 (75.0%)	1.00
Fatigue Assessment Scale	26.5 (SD = 8.0)	24.5 (SD = 5.2)	0.66
Patient Health Questionnaire-9	7.6 (SD = 4.3)	6.3 (SD = 3.0)	0.59
Nottingham Extended Activities of Daily Living	20.8 (SD = 0.9)	18.0 (SD = 2.6)	0.12
Stroke Impact Scale total score (SIS)	250.0 (SD = 31.0)	250.8 (SD = 17.9)	0.97
SIS General Rating	74.8 (SD = 16.7)	80.0 (SD = 8.2)	0.57
SIS Physical Strength	89.1 (SD = 9.9)	73.4 (SD = 10.7)	0.03*
SIS Memory and Thinking	75.4 (SD = 21.3)	77.7 (SD = 22.7)	0.88
SIS Emotion	66.0 (SD = 26.1)	81.9 (SD = 13.9)	0.20
SIS Communication	80.4 (SD = 23.3)	93.8 (SD = 7.9)	0.18
SIS Daily Activities	93.4 (SD = 6.54)	90.0 (SD = 4.1)	0.37
SIS Mobility	87.5 (SD = 15.1)	86.8 (SD = 11.4)	0.94
SIS Hand Function	93.8 (SD = 9.5)	81.3 (SD = 17.0)	0.13
SIS Social Activity	67.8 (SD = 24.5)	59.4 (SD = 35.1)	0.63

*p value < 0.05

#### Attendance

Of the eight retained participants, all attended their face-to-face sessions as planned. For the telephone-delivered booster session, only one participant was available as planned, whilst the other seven participants requested that their sessions be rearranged as they had forgotten about the appointment or were too busy to be available at the designated time.

#### Participant feedback

Feedback was obtained from the eight retained participants (five attended the feedback meeting and three gave feedback by telephone). Seven participants (87.5%) rated the intervention as ‘very useful’ or ‘somewhat useful’ (Table 3). Three participants reported that they had not used the strategies to regulate sleep or challenge thoughts, as these problems were not relevant to them. The rating of the usefulness of regulating sleep varied among participants. Regarding the other strategies, each was rated as ‘very useful’ by more than 70% of the participants who had used it. One participant rated the intervention as ‘not useful at all’. This included strategies for regulating sleep and the booster session, though interestingly the fatigue score of this participant was improved from baseline to after treatment.

**Table 3:** Participant ratings of the usefulness of the intervention (n = 8).

Intervention strategies	Numbers of participants who had used the strategy	Numbers of participants who gave each level of the rating			
		Not useful at all	A little useful	Somewhat useful	Very useful
General rating of intervention	8	1 (12.5%)	0	2 (25.0%)	5 (62.5%)
Fatigue education	8	0	0	1 (12.5%)	7 (87.5%)
Activity and sleep diaries	8	0	1 (12.5%)	1 (12.5%)	6 (75.0%)
Planning activities	8	0	2 (25.0%)	0	6 (75.0%)
Regulating sleep	5	1 (20.0%)	1 (20.0%)	1 (20.0%)	2 (40.0%)
Challenging thoughts	5	0	0	1 (20.0%)	4 (80.0%)
Overcoming setbacks	7	0	0	2 (28.6%)	5 (71.4%)
Review session	8	1 (12.5%)	1 (12.5%)	0	6 (75.0%)

More than 60% of participants rated homework tasks as ‘not difficult at all’ or ‘a little difficult’ (Table 4). Some participants reported difficulties in keeping diaries, making plans, increasing activities or regulating sleep, though they commented that these tasks were useful in helping them overcome fatigue. Five participants rated the content of sessions as being of ‘the right amount’, whilst two participants reported Session 1 was ‘too much’ and the other thought Session 6 was ‘too little’.

**Table 4:** Participant ratings of the difficulty in completing homework tasks (n = 8).

Homework tasks	Numbers of participants who had used the strategy	Numbers of participants who gave each level of the rating			
		Not difficult at all	A little difficult	Somewhat difficult	Very difficult
Keeping diaries	8	4 (50.0%)	1 (12.5%)	3 (37.5%)	0
Making plans	8	5 (62.5%)	2 (25.0%)	1 (12.5%)	0
Increasing activities	8	4 (50.0%)	1 (12.5%)	3 (37.5%)	0
Challenging thoughts	5	2 (40.0%)	3 (60.0%)	0	0
Regulating sleep	5	3 (60.0%)	0	0	2 (40.0%)

All participants had concerns about our future plans for stroke nurses to deliver this intervention. Nevertheless, they conceded that the nurse-delivery approach could be acceptable if stroke nurses were to be trained and supervised by experienced CBT psychotherapists. All participants suggested that treatment sessions and booster sessions should be delivered in person. They felt that they would be taken more seriously when meeting the therapist in person and that this would result in their greater commitment to the therapeutic process. Based on their experience of telephone-delivered booster sessions, they had had difficulty in being available at the agreed times and were easily distracted by their home environment during the telephone session. Participants also intimated that they would not wish to undertake such a therapeutic process online as it was unlikely that they would regard it as personal or as relevant as a person-to-person approach.

#### Therapist feedback

The therapist (KA), a clinical psychologist experienced in delivering CBT, commented that the intervention ‘had been carefully designed on the basis of standard CBT literature’ and that the manuals ‘had been clearly laid out’. However, she suggested a more even distribution of materials between sessions, as there was too much new information in Session 1 and insufficient material in Session 6 for some participants.

#### Clinical outcomes

Clinical outcomes of the eight retained participants are summarised in Table 5. Compared to the baseline scores, there was improvement in fatigue severity, self-reported general recovery, memory and thinking, emotion, mobility, and social activity at three months after the end of treatment (all *p*<0.05).

**Table 5:** Clinical outcomes at baseline and post-treatment assessments (n = 8).

Measures	Baseline	Post-treatment assessment		One-month assessment		Three-month assessment	
		Mean(SD)	Mean difference (95% CI)	Mean(SD)	Mean difference (95% CI)	Mean(SD)	Mean difference (95% CI)
PSF case definition	62.5%	25.0%	*p* = 0.25	25.0%	*p* = 0.25	12.5%	*p* = 0.13
FAS	26.5 (8.0)	21.8 (7.4)	4.8 (-2.1, 11.6); *p* = 0.15	19.5 (8.4)	7.0 (-0.8, 14.8); *p* = 0.07	17.3 (8.6)	9.3 (1.4, 17.1); *p* = 0.03*
PHQ-9	7.6 (4.4)	4.8 (4.9)	2.9 (-0.002, 5.8); *p* = 0.05	4.5 (5.1)	3.1 (0.2, 6.1); *p* = 0.04*	5.0 (6.3)	2.6 (-0.7, 5.9); *p* = 0.10
NEADL	20.8 (0.9)	20.5 (1.2)	0.3 (-0.3, 0.8); *p* = 0.35	20.9 (1.1)	-0.1 (-1.0, 0.7); *p* = 0.73	21.0 (1.1)	-0.3 (-1.0, 0.5); *p* = 0.45
SIS General Recovery	74.8 (16.7)	84.1 (12.6)	-9.4 (-18.8, 0.1); *p* = 0.05	84.6 (15.7)	-9.9 (-20.1, 0.4); *p* = 0.06	88.6 (15.2)	-13.9 (-25.8, -2.0); *p* = 0.03*
SIS Physical Strength	89.1 (9.9)	87.5 (13.8)	1.6 (-10.8, 14.0); *p* = 0.78	85.9 (19.7)	3.1 (-13.6, 20.0); *p* = 0.67	89.8 (12.5)	-0.8 (-14.0, 12.4); *p* = 0.90
SIS Memory and Thinking	75.5 (21.3)	79.9 (23.3)	-4.5 (-10.2, 1.2); *p* = 0.11	82.0 (24.4)	-6.6 (-15.3, 2.2); *p* = 0.12	87.1 (18.0)	-11.6 (-19.2, -4.0); *p* = 0.009*
SIS Emotion	66.0 (26.1)	76.0 (27.0)	-10.1 (-24.1, 3.9); *p* = 0.13	78.8 (22.8)	-12.8 (-27.5, 1.8); *p* = 0.08	84.4 (18.8)	-18.4 (-30.6, -6.2); *p* = 0.009*
SIS Communication	80.4 (23.3)	85.7 (23.0)	-5.4 (-16.3, 5.6); *p* = 0.29	87.5 (21.9)	-7.1 (-18.2, 3.9); *p* = 0.17	87.5 (17.9)	-7.1 (-15.9, 1.6); *p* = 0.09
SIS Daily Activities	93.4 (6.5)	92.5 (10.2)	0.9 (-4.4, 6.3); *p* = 0.69	92.8 (8.3)	0.6 (-4.7, 5.9); *p* = 0.79	95.9 (5.3)	-2.5 (-5.5, 0.5); *p* = 0.09
SIS Mobility	87.5 (15.1)	91.0 (14.1)	-3.5 (-5.5, -1.4); *p* = 0.005*	91.3 (15.0)	-3.8 (-6.8, -0.8); *p* = 0.02*	91.3 (14.0)	-3.8 (-7.1, -0.6); *p* = 0.03*
SIS Hand Function	93.8 (9.5)	93.8 (11.6)	0.0 (-4.5, 4.5); *p* = 1.00	92.5 (11.0)	1.3 (-2.5, 5.0); *p* = 0.45	95.6 (8.6)	-1.9 (-9.6, 5.8); *p* = 0.58
SIS Social Activity	67.9 (24.6)	86.3 (14.5)	-18.5 (-29.4, -7.6); *p* = 0.005*	82.8 (24.3)	-15.0 (-26.7, -3.2); *p* = 0.02*	82.4 (19.9)	-14.6 (-23.3, -5.8); *p* = 0.006*

*p value < 0.05.

FAS: Fatigue Assessment Scale; PHQ-9: Patient Health Questionnaire-9; NEADL: Nottingham Extended Activities of Daily Living; SIS: Stroke Impact Scale. 95% CI: 95% confidence interval. Mean difference: difference of scores between baseline and each post-treatment assessment (for FAS and PHQ-9 positive values indicate improvement; for other measures, negative values indicate improvement).

## Discussion

This paper describes an evidence-based process of developing a manualised psychological intervention for PSF based on the MRC framework [8]. The treatment targets were determined on the basis of clinical observation and justified by a systematic review of the literature. The intervention programme was adapted from existing psychological interventions for fatigue in health conditions other than stroke, with iterative input from stroke clinicians, psychological therapists, and stroke survivors, considering both theoretical and practical issues in stroke care and clinical psychology. This intervention was based on a cognitive behavioural therapeutic approach to challenge patients’ cognitive representation of fatigue and to encourage them to increase their daily activities. By gradually increasing their physical activity in daily living, patients were able to break the vicious cycle that perpetuate fatigue. The intervention was well received by both stroke patients and the clinical psychologist, and most participants rated it very useful in helping them overcome PSF.

In response to feedback from participants and therapist, we modified the intervention for future use by incorporating the following amendments: a) combining sessions 5 and 6 into one session; b) delivering the booster session in person rather than by telephone; and c) allowing flexibility in designing individualised programmes, as some strategies (e.g. sleep regulating) did not apply to all patients with PSF.

We screened 421 patients with the intention of securing the engagement of 12 participants. We excluded patients who were medically unstable or severely depressed, because fatigue associated with other medical conditions could be improved as these conditions were treated. We decided in advance to include patients with a PHQ-9 score between 10 and 14 (indicating moderate depression), if they did not undertake any treatment for depression. This intervention for fatigue was similar to interventions for mood, thus we anticipated improvement in both fatigue and mood, a result which was reflected in the clinical outcomes of this study.

Although the withdrawal of three participants was not directly related to this intervention (i.e. one did not attend any sessions and two others withdrew because they had become ill), the fourth participant withdrew as she reported that she did not require intervention for her fatigue and, indeed, had ‘just wished to contribute to the research’. To screen patients with PSF, we adapted a single question ‘Do you feel tired all the time or get tired very quickly since your stroke?’ from the Greater Manchester Stroke Assessment Tool (GM-SAT) [25] and provided a simple answer of ‘yes’ or ‘no’. Learning from this participant, in future trials we will expand the answers to ‘Yes and I would like additional help and support’; ‘Yes but I am receiving enough help and support’; and ‘No’, which are the original answers for this question in the GM-SAT [25].

In this feasibility study the intervention was delivered by a clinical psychologist; this delivery approach might not be possible in practice, however, because of the shortage of trained psychologists in stroke care, at least in the UK [26]. Thus, when proposing this intervention we had decided that it would ultimately be delivered by stroke nurses. We developed this intervention based on two existing psychological interventions that did not require the therapist to have specialised psychological skills [3, 13]. Based on the psychotherapist’s experience in working with nurse therapists, a short-term specialised training with regular supervision by experienced psychotherapists was deemed to be sufficient to train nurses to deliver a psychological intervention for cancer-related fatigue [3]. Whether this nurse-delivery approach is feasible for PSF will be tested in the next stage trials.

Although the statistical power of this study was limited, there was significant improvement in fatigue severity. However, this study did not have a control group, thus we do not know whether the improvement was due to the therapeutic effect or the natural resolution of fatigue. Intervention efficacy should be investigated in future RCTs. Another limitation of this study was that the feasibility of this intervention in less independent patients is unknown. All participants had good independence, with baseline NEADL scores ranging from 20 to 22 (out of a maximal score of 22 for full independence). Thus, the generalisability of this intervention in general stroke patients needs to be investigated in future studies with a more diverse stroke population.

## Conclusions

This psychological intervention was acceptable to the majority of the small group of stroke patients that we delivered it to and was feasible in the local health service. A pilot RCT would be a useful next stage to further test its feasibility and to collect data to inform definitive trials.

## Supporting information

S1 File Study protocol. A feasibility study of a brief psychological intervention for post-stroke fatigue.
(PDF)

S2 File TREND statement checklist. (PDF)
