# Supplementary material for: Development of a psychological intervention for fatigue after stroke
Source: PLoS One. 2017 Aug 17;12(8):e0183286. doi: 10.1371/journal.pone.0183286 (PMC5560529; doi:10.1371/journal.pone.0183286)
Supplement: S1 File — A feasibility study of a brief psychological intervention for post-stroke fatigue. (PDF) [file pone.0183286.s001.pdf]

Study Protocol

A feasibility study of a brief psychological intervention for post-stroke fatigue

Funders: University of Edinburgh/China Scholarship Council

Protocol Author: Dr Simiao Wu

Supervisors: Prof. Gillian Mead

Prof. Malcolm Macleod

Version 1.0

01 April 2014

Contents

List of abbreviations 4

Introduction 5

Aims and objectives 8

For intervention design 8

For trial design 8

For clinical outcomes 8

Methods 9

Trial design 9

Participants 9

Inclusion criteria 9

Exclusion criteria 9

Therapist 10

Trial process 11

Step 1: Recruitment 11

Step 2: Six therapy sessions 19

Step 3: Post-treatment assessment 21

Step 4: Assessment at one-month after treatment 21

Step 5: Interim analysis of individual data 21

Step 6: Feedback session 21

Step 7: Feedback meeting with participants 22

Step 8: Three-month follow up 22

Feedback from the therapist 22

Outcomes 22

Feasibility outcomes 23

Clinical outcomes 24

Data analysis 24

Ethics approval 25

Trial management 25

Timeline 26

List of abbreviations

CHSS	Chest Heart and Stroke Scotland
COGRAT	Cognitive Therapy with Graded Activity Training
CRF	Clinical Research Facility
FAS	Fatigue Assessment Scale
FOCUS	Fluoxetine Or Control Under Supervision
MRC	Medical Research Council
NADL	Nottingham Extended Activities of Daily Living
NHS	National Health Service
PHQ-9	Patient Health Questionnaire-9
PSF	Post-stroke Fatigue
RCT	Randomised Controlled Trial
RIE	Royal Infirmary of Edinburgh
SIS	Stroke Impact Scale
WGH	Western General Hospital

Introduction

Stroke is a major cause of mortality and morbidity worldwide. Fatigue is a common symptom after stroke, which affects about 30-70% of stroke survivors [1]. Post-stroke fatigue usually occurs at an early stage after stroke and could be persistent in the long term [2]. Previous studies indicated that post-stroke fatigue is associated with functional dependency in daily life and a higher risk of institutionalisation, and also contributes to a higher rate of death [3]. For both stroke survivors and clinical professionals, fatigue ranks among the top ten research priorities relating to life after stroke [4].

In spite of the high prevalence and detrimental consequences of post-stroke fatigue, its mechanisms are unknown and there is no recommended treatment [5]. Although some patients think their fatigue was a result of their stroke or brain recovery [6], studies found no association between post-stroke fatigue and stroke type, severity or time after stroke. The association between post-stroke fatigue and lesion site is inconclusive [7, 8]. Post-stroke fatigue is a complex phenomenon associated with physical, psychosocial and behavioural factors [9] and psychological interventions targeting these factors have improved fatigue in patients with other conditions, such as multiple sclerosis [10], cancer [11], and chronic fatigue syndrome [12]. To provide evidence for adapting similar interventions for post-stroke fatigue, we performed a systematic review of studies reporting psychological associations of post-stroke fatigue. This review found that depressed mood, anxiety, loss of control, and inadequate coping styles, as well as emotional and behavioural problems are all associated with post-stroke fatigue.

A randomised controlled trial (RCT), the COGRAT study, has compared a combined therapy of psychological intervention and exercise training with the psychological intervention alone [13], and reported that the combined therapy was more effective than the psychological intervention. Based on currently available evidence, there are some research gaps for guiding treatment for post-stroke fatigue. Firstly, a multicentre RCT has reported that this type of psychological intervention is cost effective compared with either exercise training or usual care, in improving chronic fatigue syndrome [14]. But we do not know if this is the case for post-stroke fatigue. Secondly, the COGRAT study only included patients with severe fatigue according to a fatigue scale; however, as fatigue is a common symptom after stroke, it would be helpful to test the intervention in a wider range of stroke survivors, that is, anyone takes fatigue as a significant concern after their stroke. Thirdly, in previous studies, the psychological intervention for post-stroke fatigue was all delivered by psychologists. However, psychologists are usually not involved in routine care for stroke survivors within the National Health Service (NHS). Thus it is better to adapt the intervention to be delivered by stroke nurses who provide routine health service for stroke survivors. Finally, the existing psychological intervention for post-stroke fatigue is group-based, whereas the home visiting by nurses is usually on a one-to-one basis. Thus an individually delivered therapy might be more feasible in practice.

With the above concerns, we have developed a brief psychological intervention for post-stroke fatigue. Regarding the delivery process, this is a 'brief' intervention as it is a short-term intervention that aims to address a specific concern in a few treatment sessions. Regarding the therapeutic components, this is a 'complex' intervention that contains several interacting components and the complexity may arise through several dimensions. The *Medical Research Council (MRC) framework of design and evaluation of complex interventions to improve health* [15] has suggested a five-stage approach for the development:

Phase 0: Theoretical phase (why should this intervention work?)

Phase 1: Modelling phase (how does it work?)

Phase 2: Exploratory phase (optimising trial measures)

Phase 3: Explanatory phase (definitive randomised controlled trial)

Phase 4: Implementation (apply to clinical practice)

The MRC framework also suggested researchers consider phases 0, 1, and 2 as part of one larger iterative activity rather than as sequential stages [16]. Therefore, this feasibility study is part of both phase 1 and phase 2 studies that involves a mixture of qualitative and quantitative approaches.

The effectiveness of this psychological intervention in improving fatigue in stroke survivors will be determined by a definitive randomised controlled trial (RCT, phase 3) in control with the usual care. To inform such a large phase 3 trial, two preliminary trials will be conducted to determine the feasibility of the intervention and the trial design. **In the current study**, a feasibility study (phase 1 and part of phase 2) is being conducted to determine the content of the intervention and whether it is acceptable to both patients and therapist, as well as test the recruitment and follow up processes. If the intervention is acceptable and relevant trial processes are feasible, a pilot RCT (phase 2) will be conducted to determine the randomisation process, and to obtain data on variability of outcome measures to inform power calculation for the definitive trial. The comparison of the trials of phases 1, 2 and 3 is summarised in Table 1.

Table 1 A summary of the three-stage trials for the development of a brief psychological intervention for post-stroke fatigue

<table>
<tr><th>Phases</th><th>Stages of development</th><th>Aims</th><th>Trial design</th></tr>
<tr><td>Phase 1 (modelling phase)</td><td rowspan="2">Feasibility trial (current study)</td><td rowspan="2">1) To determine the acceptability and feasibility of the intervention
2) To determine the feasibility of the recruitment process, follow up process, and outcome measures</td><td rowspan="2">Single centre;
Single-arm no control;
Delivered by a psychologist;
Sample size N=12</td></tr>
<tr><td rowspan="2">Phase 2 (exploratory phase)</td></tr>
<tr><td>Pilot RCT</td><td>3) To test whether the trial design is feasible for a RCT (including randomisation process)
4) To obtain data to inform power calculation for the definitive trial</td><td>Single centre;
Usual care control;
Delivered by a stroke nurse;
Sample size N=60</td></tr>
<tr><td>Phase 3 (explanatory phase)</td><td>Definitive RCT</td><td>5) To investigate the effectiveness and safety of the intervention</td><td>Multiple centres;
Usual care control;
Delivered by stroke nurses;
Sample size depends on the results of the pilot RCT</td></tr>
</table>

Aims and objectives

According to the MRC framework [15], the aim of this feasibility study is to define the intervention and the trial design.

The primary objectives of this study are:

For intervention design

1. To determine whether a psychological intervention is acceptable to patients with post-stroke fatigue
2. To determine whether this intervention is acceptable to a therapist who has a psychological background
3. To determine how to adapt this intervention to be delivered by stroke nurses who do not have a psychological background

For trial design

4. To test the feasibility of recruitment process
5. To determine the response rates of three-month follow up and missing data

For clinical outcomes

6. To test the feasibility of completing questionnaires for clinical outcomes

The secondary objective is to perform a before-and-after analysis of clinical outcomes.

1) Fatigue, measured by the Fatigue Assessment Scale (FAS) and by a Case Definition of post-stroke fatigue
2) Depression, measured by Patient Health Questionnaire-9 (PHQ-9)
3) Independence in daily life, measured by Nottingham Extended Activities of Daily Life (NADL)
4) Stroke-specific health-related quality of life, measured by Stroke Impact Scale (SIS)

Methods

Trial design

This is a single-arm trial designed to assess the feasibility of the intervention and trial design. The intervention will be delivered at a single medical centre by a clinical psychologist (the therapist).

Participants

A total of 12 participants will be recruited. Patients who meet all of the following inclusion criteria and do not have any of the exclusion criteria will be eligible for the trial. .

Inclusion criteria

1) Post-stroke fatigue

Patients will be asked by the question ‘Do you feel tired all the time or get tired very quickly since your stroke?’ This question is part of the Greater Manchester Stroke Assessment Tool, which identified fatigue as the most frequent (34.3%) unmet need in 137 community stroke survivors [17]. In the current study, stroke survivors who answer ‘Yes’ to this question will be taken as having post-stroke fatigue.

2) Between three months and two years after stroke onset

Stroke survivors within this time period are likely to have persistent fatigue, to be medically stable, and to remain in the most active rehabilitation and recovery period. We will also include patients with minor stroke, but not transient ischaemic attack (TIA).

3) Over 18 years of age

4) Live in local areas of Lothian

Exclusion criteria

1) Severe depression

Depression will be assessed by the Patient Health Questionnaire-9 (PHQ-9), of which a total score of 15 or more would indicate severe depression. We will exclude patients with a total score of PHQ-9 of 15 or more. This is because there is a large overlap between symptoms of fatigue and depression. Fatigue accompanying post-stroke depression is often relieved when the depression is adequately treated.

2) Significant impairments that preclude them from being benefit from the intervention, for example:

- Severe cognitive deficits (This would preclude them from fully understanding the intervention materials)
- Severe aphasia (Participants need to be able to carry out effective conversation with the therapist)
- Other significant difficulties in verbal communication

3) Medically unstable in the opinion of a medical clinician (This will be determined by contacting their General Practitioners)
4) Have another unfavourable condition that could impact results (e.g. substance abuse)
5) Currently in the nursing home
6) Currently in other research studies that might affect fatigue or add significant burden to the participant, for example, studies have outcome measures for fatigue or involve physical training (e.g. the FOCUS study)

Multiple stages will be taken to determine the eligibility for participation, including:

- Screening the discharge summary stored in the TRAK system, which is the electronic database of the NHS (e.g. for age, time after stroke, settlement in Lothian)
- Contacting the General Practitioners (GP) of the potential participants (e.g. for current depression, communication capacity, medical stability, unfavourable condition, whether in other research studies)
- Reviewing the results of survey questionnaire completed by the potential participants (e.g. for post-stroke fatigue and depression)
- Telephone conversation and face-to-face meeting between the potential participants and the researchers (e.g. for capacity of cognition and communication)

Therapist

In this feasibility study, the intervention will be delivered by a clinical psychologist (Dr Kirsty Anderson), who has the experience in delivering psychological interventions as well as working with stroke survivors. This psychologist-delivered approach will help to standardise the design of the psychological intervention.

If the current study shows the intervention is acceptable to both stroke survivors and the psychologist, the intervention will be adapted to be delivered by stroke nurses in subsequent trials (e.g. the definitive trial for the effectiveness and safety). In a study comparing the therapist effect on treatment outcomes of a similar psychological intervention for chronic fatigue syndrome, there was no difference between psychologists

and nurses, given adequate training and supervision provided [18]. Therefore, in this feasibility study, we will collect information from both the therapist and participants to adapt this intervention to be delivered by stroke nurses.

Trial process

Step 1: Recruitment

There will be a **two-stage recruitment** process for this study, i.e. the first stage of a survey for fatigue (Figure 1) and the second stage of a trial for fatigue (Figure 2).

At the first stage, we will identify potential participants by **three approaches**, i.e. from the Stroke Ward of Royal Infirmary of Edinburgh, from the Stroke Clinic of Western General Hospital, and from community by the CHSS Stroke Nurses. This is to ensure that the first contact to potential participants is from the members of their direct medical care teams.

For each identification approach, we will have **three subsequent processes** to assess the eligibility of potential participants, i.e. by reviewing the medical records and contacting GPs, by questionnaires, and by a face-to-face meeting.

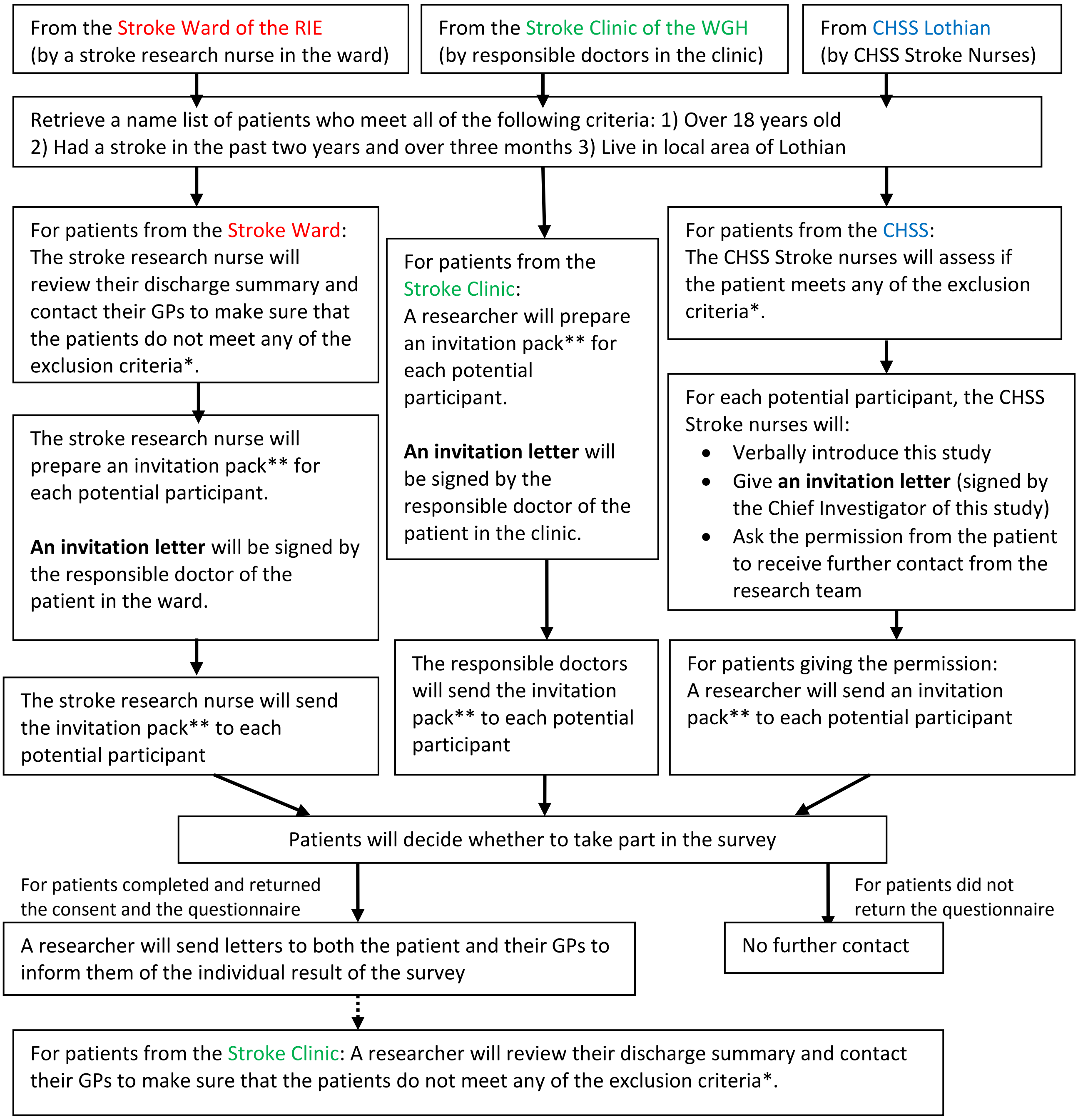

Figure 1 The first stage of recruitment: a survey for post-stroke fatigue

*Exclusion criteria:

- Severe cognitive deficits, sever aphasia, or significant difficulty in communication
- Medically unstable or in other unfavourable conditions (e.g. substance use)
- Currently in a nursing home
- Currently in other research studies that might affect fatigue

**The invitation pack contains:

- An information sheet of the survey
- Consent to complete the survey, to be contacted by the research team, to allow researchers to access their medical records, and to give permission that their GPs be informed of their participation
- A questionnaire for the survey of post-stroke fatigue and depression

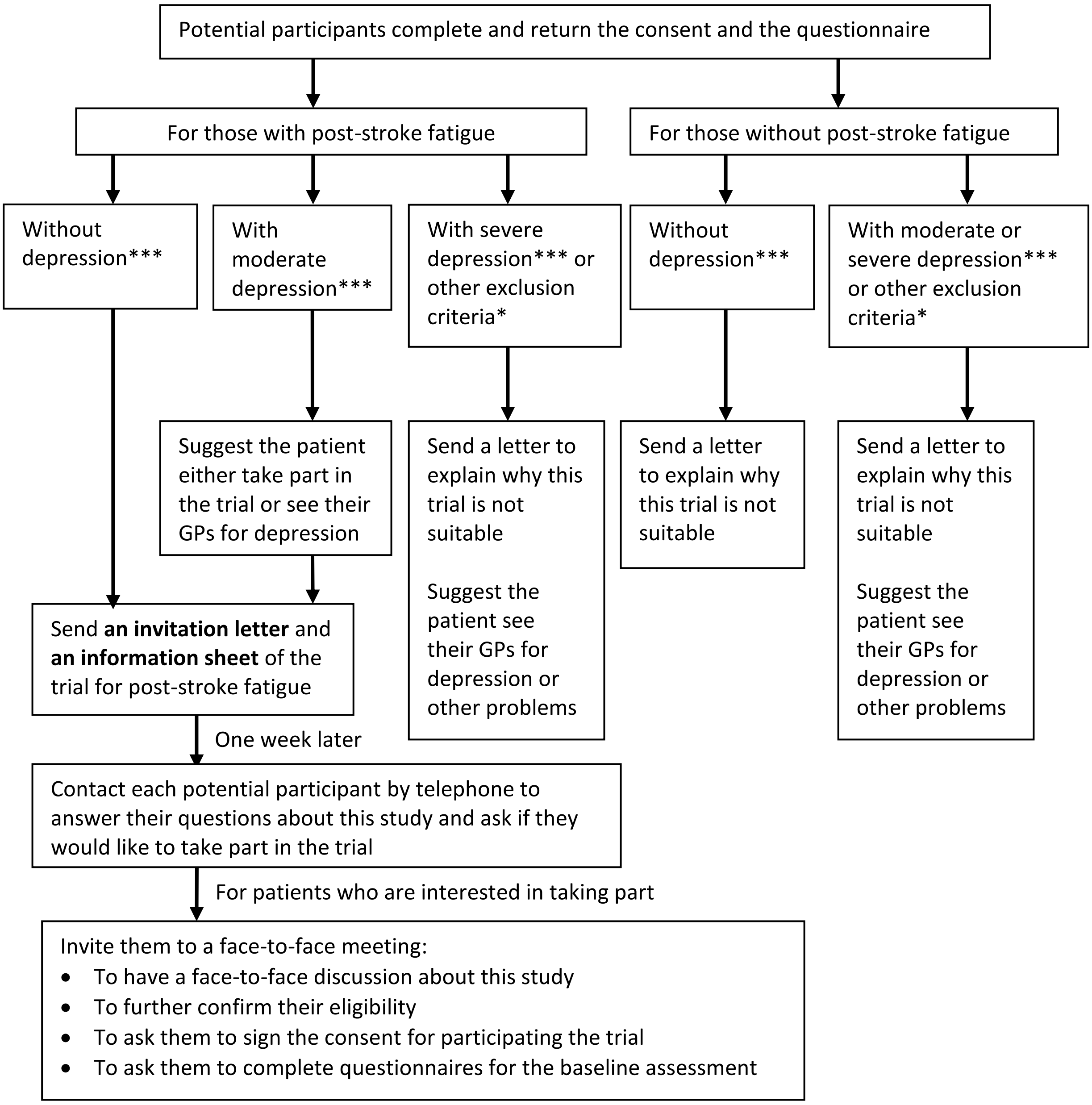

Figure 2 The second stage of recruitment: a trial for post-stroke fatigue
With permission from potential participants in the first stage, the second stage recruitment will be performed by Dr Simiao Wu, who is the Chief Investigator of this study.

*Exclusion criteria:

- Severe cognitive deficits, sever aphasia, or significant difficulty in communication
- Medically unstable or in other unfavourable conditions (e.g. substance use)
- Currently in a nursing home
- Currently in other research studies that might affect fatigue

***Depression measured by PHQ-9:

- No depression: with a total score of PHQ-9 of 9 or less
- Moderate depression: with a total score of PHQ-9 between 10 and 14
- Severe depression: with a total score of PHQ-9 of 15 or more

<u>Stage 1: A survey for post-stroke fatigue</u>

We plan to approach 100 stroke survivors to recruit 12 participants, by supposing a response rate of 50% by the initial contact (the survey for fatigue), 30% of them would be eligible for the trial, and 80% of these eligible people would agree to participate.

Approach 1: from Stroke Ward of Royal Infirmary of Edinburgh

A stroke research nurse, who is the member of the direct medical care team of stroke survivors in the stroke ward, will help to identify potential participants for this study.

1) Initial assessment of eligibility before contacting stroke survivors

The stroke research nurse will review the ward records to obtain a name list of potential participants who meet the following inclusion criteria:

1) Had a stroke in the past two years and over three months after stroke
2) Over 18 years old
3) Live in the local area of Lothian

Then the nurse will review the NHS TRAK system for the discharge summary of the listed potential participants and contact their Family doctors/General Practitioners (GPs) to check that these people are **still alive** and that they have not developed any condition that would preclude them being benefit from this study. Such exclusion criteria include:

- Severe cognitive deficits
- Severe aphasia
- Significant difficulty in communication
- Medically unstable (e.g. any severe illness or injury) or in other unfavourable conditions (e.g. substance abuse)
- Being in the nursing home
- Being in other research studies that might affect fatigue or add significant burden to the participants

2) Further assessment of eligibility by a survey for post-stroke fatigue and depression

For potential participants who meet all of the above inclusion criteria and do not have any of the exclusion criteria, they will receive an invitation pack which provides the information of a survey for post-stroke fatigue and invites them to complete the survey.

The invitation pack will contain the following items:

1) An invitation letter to the survey (signed by the responsible doctor of the stroke survivor in the Stroke Ward)
2) An information sheet about the survey
3) A consent form
4) A questionnaire for post-stroke fatigue and depression

Potential participants who are willing to take part in this survey will be asked to sign the consent and complete the questionnaire, and return them to the research team using a stamped addressed envelope.

By signing the consent form, the participants will:

- Give consent to take part in the survey (to complete the questionnaire)
- Give permission to the research team to access their medical records
- Give permission to receive further contact from the research team
- Allow their GPs to be informed of their participation and their results of survey

Approach 2: from Stroke Clinic of Western General Hospital

This recruitment process from the stroke clinic is similar with that from the stroke ward. The key differences between the two approaches are summarised as follows.

The responsible doctors in the stroke clinic will review the clinical records to obtain a name list of potential participants who meet the following inclusion criteria:

1) Had a stroke in the past two years and over three months after stroke
2) Over 18 years old
3) Live in the local area of Lothian

A researcher will prepare an invitation pack of the survey for post-stroke fatigue for each potential participant. The responsible doctors will then sign the invitation letters and send them to potential participants. Only after receiving the signed consent from the participants will the researcher review their medical records and contact their GPs to further check their eligibility. The following assessment process is the same as that of the Approach 1.

Approach 3: from community through the CHSS Lothian

This recruitment process from the stroke clinic is similar with that of the other two approaches. The key differences are summarised as follows.

The CHSS Stroke nurses who routinely visit stroke survivors in community will help to identify potential participants who meet all of the inclusion criteria and do not have any of the exclusion criteria. For stroke survivors who might be suitable for this study, the CHSS nurses will:

- Verbally introduce this study to potential participants
- Give an invitation letter to them (signed by the Chief Investigator of this study)
- Ask for their permission to receive further contact from the research team

With the permission from potential participants, a researcher will send them an invitation pack to invite them to take part in the survey for post-stroke fatigue. The following assessment process is the same as that of the Approach 1.

Stage 2: A trial for post-stroke fatigue

For potential participants who return the questionnaire and give the consent, a researcher will look at their questionnaires to check their eligibility. As shown in Table 2, the researcher will apply the criteria step by step (reading the Table from left to right) to determine 1) whether the participants meet the criteria for post-stroke fatigue, and 2) whether they have post-stroke depression, and if so, how severe it is. Based on this result, the researcher will determine whether the patient is eligible for further assessment.

Table 2 Screening for post-stroke fatigue and depression

Question for **post-stroke fatigue:**<br>"Do you feel tired all the time or get tired very quickly since your stroke?"	Questionnaire for **depression:**<br>Total score of PHQ-9	**Conclusion:**<br>Eligible for further assessment?
'Yes'	<=9	**Eligible**
	Between 10 and 14	**Eligible**
	>=15	Ineligible
'No'	<=9	Ineligible
	>=10	Ineligible

The researcher will write a letter to each participant to inform them of the individual result of the survey. The researcher will also send a letter to inform their GP practice of their participation and the result of the survey (the consent for doing this will have been obtained from the participant in the consent form for the survey).

- For participants who have post-stroke fatigue and have a total score of PHQ-9 of 9 or less, we will send them an invitation letter and an information sheet to invite them to take part in a trial of a brief psychological intervention for post-stroke fatigue.
- For participants who have post-stroke fatigue and have a total score of PHQ-9 between 10 and 14, we will send them an invitation letter and an information sheet to invite them to the trial. We will suggest them try the trial first as it might be helpful for both fatigue and depression. If they are not interested in this trial, we will suggest they see their GPs to provide other health care for depression.
- For participants who have post-stroke fatigue and have a total score of PHQ-9 of 15 or more, we will not invite them to the trial and will suggest them see their GPs to provide health care for depression.

- For participants who do not have post-stroke fatigue, we will not invite them to the trial. If they have a total score of PHQ-9 of 10 or more, we will suggest them see their GPs to provide health care for depression.

One week after sending out the invitation letter and information sheet of the trial to potential participants, a researcher will contact them by telephone 1) to answer any questions that they may have, and 2) to ask if they would like to take part in the trial.

For patients who are interested in taking part in the trial or would like to learn more about this study, the researcher will invite them to a face-to-face meeting at the Clinical Research Facility (CRF) of the Royal Infirmary of Edinburgh (RIE).

3) Final assessment and consent for participation

At this meeting, each potential participant will meet the Chief Investigator of this study and a research nurse based at the CRF of RIE. The researcher will discuss with the potential participant any questions them may have about the study. The CRF nurse will help to check if this potential participant has adequate capacity in cognition and communication. This may identify a few people who are not eligible for the trial. For those patients, the researcher will explain why this trial is not suitable for them. Potential participants who are eligible for this trial and willing to take part will be asked to sign a consent form for taking part in the trial. This consent form will also be signed by the researcher and the CRF nurse.

After signing the consent, the participant will be asked to complete four of questionnaires for baseline assessment for the following clinical outcomes:

- Fatigue: Fatigue Assessment Scale (FAS) and a case definition for post-stroke fatigue
- Independence in daily life: Nottingham Extended Activities of Daily Living (NADL)
- Stroke-specific health-related quality of life: Stroke Impact Scale (SIS)

Clarification of the questionnaires

If any item of questionnaires is unanswered or appears inconsistent with the other answers, or if the participants give inappropriate answers to the questions of the case definition, the researcher will clarify the reasons with the participant.

Finally, the participant will be asked to choose a date from a list of dates for the first therapy session (the list is provided by the therapist before this meeting).

For each participant, the researcher will send a letter to inform their GPs that they are taking part in this trial for post-stroke fatigue.

Step 2: Six therapy sessions

Participants will be invited to the Department of Psychology at the Astley Ainslie Hospital or the Clinical Research Facility at the Royal Infirmary of Edinburgh for therapy sessions. The intervention consists of six face-to-face sessions. A clinical psychologist, who is the therapist of this intervention, will provide support for the participant throughout the trial. During these sessions, the participant will discuss their fatigue-related problems with the therapist and work on a treatment manual to learn skills to overcome their fatigue. Each session will last about one hour and the first session will have an extra 30 minutes at the beginning to engage the participant in a collaborative therapeutic relationship with the therapist. There will be an interval of two weeks between sessions. During the intervals, the participant will be required to do some tasks at home, including keeping a diary, carrying out an activity plan, or reading sections of the treatment manual.

The intervention incorporates both psychological and rehabilitative approaches to challenge participants' cognition and behaviours in response to fatigue. With support from the therapist, the participant will explore their own problems in thoughts (their beliefs about fatigue and their ability to overcome it), emotions (their emotional responses to fatigue-related problems), and behaviours (physical activities in daily life) which may potentially contribute to their fatigue. Then they will work together to find out individualised treatment strategies to help the participant reduce the impact of fatigue on their daily life. The therapeutic components of the intervention, as well as the aim, rationale and expected outcomes for each component are summarised in Table 3.

Table 3 A summary of the intervention

Therapeutic components	Aim	Rationale	Expected outcomes
Education about post-stroke fatigue	To provide individualised treatment rationale	Post-stroke fatigue is a complex symptom that affects physical, emotional, cognitive and behavioural functions;<br><br>The impact of fatigue on life is temporary and reversible;<br><br>Building up confidence to overcome fatigue is very important for this psychological therapy	Participants will understand their own problems that contribute to fatigue;<br><br>Participants will regain self-confidence in overcoming fatigue;<br><br>Participants will be willing to engage in this treatment therapy
Regulating the sleep pattern	To develop a regular pattern of sleep	It is common for stroke survivors to have problems of sleep;<br><br>Disturbed sleep is a common cause for the feeling of tiredness	Participants will develop a regular pattern of sleep with adequate amount of time for sleep each night, a fixed getting up time, and avoiding naps during the day
Graded activity programme	To gradually increase physical capacity	Adaptation of activity patterns to current energy level;<br><br>Balancing daily activities, rest and sleep;<br><br>Gradually increasing the amount of physical activity doing each day	Participants will develop a more active pattern of daily life, with different activities spread over the week and balanced with planned rests during the day
Challenging unhelpful thoughts and emotions	To identify and deal with any emotions or thoughts that block the progress	Thoughts, emotions and behaviours are interacted with each other and are contributing to fatigue symptoms	Participants will be aware of unhelpful thoughts and emotions and will be able to act in a more positive and effective pattern
Managing blocks and setbacks	To identify and deal with (potential) difficulties in making progress	It is understandable that people will have difficulties in changing the way they used to think and act	Participants will be prepared for potential problems and will be able to manage, should they occur

Step 3: Post-treatment assessment

At the end of the last face-to-face session, each participant will take home five questionnaires for post-treatment assessment, including:

- Fatigue Assessment Scale (FAS)
- A case definition of post-stroke fatigue
- Patient Health Questionnaire-9 (PHQ-9)
- Nottingham Instrumental Activity of Daily Life (NADL)
- Stroke Impact Scale (SIS)

The participant will be asked to complete these questionnaires at home and return to the research team within a week. If the questionnaires have not been returned within two weeks, the researcher will contact the participant by telephone to remind them and, if necessary, complete the questionnaires with the participant through the telephone.

Step 4: Assessment at one-month after treatment

One month after the last face-to-face session, the researcher will post to each participant a same set of questionnaires as used for post-treatment assessment and a questionnaire for their feedback on their experience of the trial. The Participant Feedback Form includes both specific questions (intensity/workload, frequency and duration of the intervention) and non-specific questions (any questions raised by participants regarding the trial). The participant will be asked to complete and return the questionnaires within a week. If the questionnaires have not been returned within two weeks, the researcher will contact the participant by telephone to remind them and, if necessary, complete the questionnaires with the participant through the telephone.

Step 5: Interim analysis of individual data

After receiving the completed questionnaires for one-month assessment, the researcher will analyse the results of individual participant for their baseline assessment, assessment immediately after treatment, and assessment at one-month after treatment. Both participants and the therapist will receive the individual result in mailing letters.

Step 6: Feedback session

Within one week receiving the result of the interim analysis for individual participant, the therapist will contact the participant by telephone to deliver a feedback session. This telephone-delivered session will include the following content:

1) Discussing the results of the interim analysis. Informing participants of the results is part of the intervention. If participants have made improvement in clinical outcomes, this will encourage them to continue using the skills that they feel useful.

2) If there is no significant improvement after treatment or the participant could not maintain improvement in the past one month, potential problems and possible solutions will be discussed.

3) Discussing the skills that the participant continued to use during the past month and clarify reasons for not using certain planned skills.

4) Checking participants' understanding of this therapy and clarify any unclear aspects.

5) Discussing how to make further improvement and making a plan.

Step 7: Feedback meeting with participants

After all individual feedback sessions completed, we will invite all of our 12 participants to a group meeting. This meeting will be at the Clinical Research Facility of Royal Infirmary of Edinburgh and a researcher will facilitate this meeting. During this meeting, we will invite our participants to share their experiences of taking part in this trial and to make suggestions for how their experiences of the trial, and of the therapy, could be improved. We will also explain that this intervention will be adapted to be delivered by nurses who visit stroke survivors in community, and will ask participants for their opinions on how this.

Step 8: Three-month follow up

Three months after the therapy sessions, the researcher will send each participant again the same set of questionnaires. This is to assess whether any gains the participant may have made could be retained and whether any further improvement has been made. The researcher will also write to each participant to inform them of the individual result of this assessment.

Feedback from the therapist

After all the telephone-delivered feedback sessions, the Chief Investigator and the therapist will have a feedback meeting to discuss problems occurring during the intervention delivery and possible solutions. They will also discuss how to adapt this intervention to be delivered by stroke nurses.

Outcomes

A combination of qualitative and quantitative measures will be used to address the research questions relating to the acceptability of the intervention and the feasibility of conducting a definitive trial in the future.

Feasibility outcomes

1. Acceptability of intervention to therapist

   - Consistency of therapist delivering the intervention

     For each individual therapy session, we will provide a **Checklist of Session Content** to the therapist. This checklist contains important therapeutic components to be covered during each session. The therapist will also take notes for each session to record what has been discussed during the session and how cooperative the participant is during and between the sessions.

2. Acceptability of intervention to participants

   - Feedback meeting after the feedback session
   - Participant Feedback Form (completed by the participant one month after the last face-to-face session)

3. Feasibility of trial process

   1) Recruitment

   - Number of discharge summary/clinical records/community survivors screened
   - Number of survey questionnaires sent out
   - Number of survey questionnaires completed and returned
   - Number of people eligible for the trial
   - Number of people consenting to participate the trial
   - Reasons for non-participation and ineligible at each stage
   - Comparison of recruitment approaches based on the stroke ward, stroke clinic and through the CHSS

   2) Therapy sessions

   - Number of sessions each participant attended and reasons for reschedule or not attending
   - Number of participants completing all sessions and reasons for withdrawal

     An **Outline of Sessions** will be used to monitor attendance of each participant at treatment sessions. This form will be completed by the therapist for each

session to record the attendance, reasons for non-attendance, and withdrawal from the treatment.

Rate of participants completing the six treatment sessions will be calculated. If any participants withdraw from the treatment sessions, we will ask them for reasons (although they can withdraw at any time without giving any reason).

3) Three-month follow up

- Number of participants completing and returning questionnaires and reasons for not completing

4. Feasibility of assessment for clinical outcomes (for baseline assessment)

   - Number of missing items of each questionnaire
   - Reason for not or inadequate completing of certain items

5. Feasibility of the nurse-led delivery

   - Feedback from both the participants and therapist on how to adapt this intervention to be delivered by stroke nurses

Clinical outcomes

Clinical outcomes will be assessed before treatment, immediately after treatment, one-month and three-month after treatment, respectively, including the following measures:

- Fatigue: **Fatigue Assessment Scale** and a **case definition for post-stroke fatigue**
- Depression: **Patient Health Questionnaire-9**
- Independence in daily life: **Nottingham Extended Activities in Daily Living**
- Stroke-specific health-related quality of life: **Stroke Impact Scale**

Data analysis

Descriptive statistics will be used to analyse the results. An interim analysis will be performed at one-month after the last face-to-face session. The final analysis will be performed at the end of the trial when all data collection, entry and validation are completed (three-month after the last face-to-face session). The data of feasibility will be analysed of a group of 12 participants. The clinical data will be analysed both individually and by group.

The number of participants attending, missing, and withdrawal (from therapy sessions and/or follow up) will be recorded and the relevant rates will be calculated. The success of

recruitment strategies will be measured by summarising responding, eligibility, and consent rated. The feasibility of assessment for clinical outcomes will be determined by the level of missing data of individual items and for the entire outcome questionnaires.

To investigate changes in clinical outcomes, we will report mean change in scores of each questionnaire with 95% confidence interval (CI) from pre-treatment assessment to post-treatment assessment. In addition, the change of scores for each clinical outcome from post-treatment to one-month follow up, and from post-treatment to three-month follow up, will be analysed to determine whether any benefits (if applicable) acquired during the therapy sessions could be maintained one month and three months after the last face-to-face session, respectively.

Ethics approval

Trial materials will be reviewed by the South East Scotland Research Ethics Committee, from which an ethics approval will be applied.

A trial sequence number will be allocated to each participant at the beginning of the trial. Identifiable information of participants will be kept on paper in a locked filling cabinet and linked to a trial sequence number. Data retrieved from the trial will be kept in link with the trial sequence number and separate from any identifiable information. All information collected during the course of the study will be kept securely (on paper and electronically) in the Department of Geriatric Medicine of Royal Infirmary of Edinburgh. All the information will be transferred and archived in compliance with the 1998 Data Protection Act.

Trial management

Dr Simiao Wu is the Chief Investigator in charge of the overall management of the study, including coordinating with members of the researcher team, promoting the study, collecting of data from participants at different stages of the study, as well as analysing, interpreting, and reporting the results.

Prof. Gillian Mead and Prof. Malcolm Macleod, both consultant neurologists, will supervise the trial and provide scientific advice throughout the study. Dr David Gillespie, a consultant psychologist, will provide supervision on the delivery process of this brief psychological intervention.

The Sponsor of the study, University of Edinburgh and NHS Lothian, will provide independent oversight of the study to ensure the study is conducted in accordance with good clinical practice.

Timeline

**Date**	**Activities**	**Duration**
January to December 2013	Development of the intervention	12 months
January to February 2014	Writing protocol	2 months
March to May 2014	Application for Ethics approval, R&D approval, and Research Passport	3 months
June to August 2014	Recruitment of 12 participants	3 months
2nd June to 30th November 2014	Six therapy sessions	26 weeks
Up to November 2014	Assessment immediately after treatment	1 month
Up to December 2014	Assessment one month after treatment	1 month
December 2014	Interim data analysis	1 month
January 2015	Feedback session	1 month
February 2015	Feedback meeting with 12 participants<br>Feedback meeting with the therapist	1 month
March 2015	Three-month follow up	1 month
April to July 2015	Data analysis and report writing	4 months

References

1. Lerdal, A., et al., *Poststroke fatigue--a review.* Journal of Pain & Symptom Management, 2009. **38**(6): p. 928-949.

2. Duncan, F., S. Wu, and G.E. Mead, *Frequency and natural history of fatigue after stroke: A systematic review of longitudinal studies.* Journal of Psychosomatic Research, 2012. **73**(1): p. 18-27.

3. Glader, E.-L., B. Stegmayr, and K. Asplund, *Poststroke fatigue: a 2-year follow-up study of stroke survivors in Sweden.* Stroke, 2002. **33**(5): p. 1327-33.

4. Pollock, A., et al., *Top ten research priorities relating to life after stroke.* The Lancet Neurology, 2012. **11**(3): p. 209.

5. McGeough, E., et al., *Interventions for post-stroke fatigue.* Cochrane Database of Systematic Reviews, 2009(3).

6. Barbour, V.L. and G.E. Mead, *Fatigue after stroke: the patient's perspective.* Stroke research and treatment, 2011. **2012**.

7. Ingles, J.L., G.A. Eskes, and S.J. Phillips, *Fatigue after stroke.* Archives of physical medicine and rehabilitation, 1999. **80**(2): p. 173-178.

8. Kutlubaev, M.A., F.H. Duncan, and G.E. Mead, *Biological correlates of post-stroke fatigue: A systematic review.* Acta Neurologica Scandinavica, 2012. **125 (4)**: p. 219-227.

9. Michael, K.M., J.K. Allen, and R.F. Macko, *Fatigue after stroke: relationship to mobility, fitness, ambulatory activity, social support, and falls efficacy.* Rehabilitation Nursing, 2006. **31**(5): p. 210-217.

10. Thomas, S., et al., *A pragmatic parallel arm multi-centre randomised controlled trial to assess the effectiveness and cost-effectiveness of a group-based fatigue management programme (FACETS) for people with multiple sclerosis.* Journal of Neurology, Neurosurgery & Psychiatry, 2013.

11. Armes, J., et al., *A randomized controlled trial to evaluate the effectiveness of a brief, behaviorally oriented intervention for cancer ‐ related fatigue.* Cancer, 2007. **110**(6): p. 1385-1395.

12. White, P.D., et al., *Comparison of adaptive pacing therapy, cognitive behaviour therapy, graded exercise therapy, and specialist medical care for chronic fatigue syndrome (PACE): a randomised trial.* The Lancet, 2011. **377**(9768): p. 823-836.

13. Zedlitz, A.M.E.E., et al., *Cognitive and Graded Activity Training Can Alleviate Persistent Fatigue After Stroke A Randomized, Controlled Trial.* Stroke, 2012. **43**(4): p. 1046-1051.

14. McCrone, P., et al., *Adaptive Pacing, Cognitive Behaviour Therapy, Graded Exercise, and Specialist Medical Care for Chronic Fatigue Syndrome: A Cost-Effectiveness Analysis.* PLoS ONE, 2012. **7**(8): p. e40808.

15. Campbell, M., et al., *Framework for design and evaluation of complex interventions to improve health.* BMJ: British Medical Journal, 2000. **321**(7262): p. 694.

16. Campbell, N.C., et al., *Designing and evaluating complex interventions to improve health care.* BMJ: British Medical Journal, 2007. **334**(7591): p. 455.

17. Rothwell, K., et al., *Feasibility of assessing the needs of stroke survivors after six months using the GM-SAT.* Clinical Rehabilitation, 2013. **27**(3): p. 264-271.

18. Cella, M., et al., *Therapist effects in routine psychotherapy practice: An account from chronic fatigue syndrome.* Psychotherapy Research, 2011. **21**(2): p. 168-178.
